# Recommendations for Resuming PA after Prolonged Rest in Children and Adolescents: A Systematic Integrative Review of Relevance for Immunity

**DOI:** 10.3390/jfmk7020047

**Published:** 2022-06-02

**Authors:** Antonio Cicchella

**Affiliations:** 1 International College of Football, Shanghai Tongji University, 1239 Siping Road, Shanghai 200092, China; antonio.cicchella@unibo.it; 2 Department for Quality-of-Life Studies, University of Bologna, Corso d’Augusto 237, 47921 Rimini, Italy

**Keywords:** physical activity, children, adolescent, immune system, training

## Abstract

This systematic integrative review aims to summarize the protective effect of PA on children and adolescents, with special reference to the immune system. Periods of prolonged inactivity in children and adolescents are rare and due to exceptional events, such as illness or environmental circumstances, e.g., natural disasters, wars, or epidemics. The recent COVID-19 pandemic forced billions of children in developmental ages into inactivity. This exceptional event was the reason for studying the compensational behavioral strategies adopted by children and adolescents to counteract physical inactivity. Several studies showed the rise of spontaneous physical activity (PA) among children and adolescents to compensate for sedentarism. However, for some children, sedentarism could in turn foster other sedentarism. With the restart of “normal daily life” worldwide, a question is posed on both how to resume PA without causing damage and how to improve the immune response. Some key points emerged from the literature. Children must resume PA gradually using different methods, considering age, sex, health status, and the presence of overweight conditions. Immunity can be stimulated with PA by aerobic exercise, resistance training, flexibility exercise, relaxation, and coordinative exercises.

## 1. Introduction

Although it is intuitive (and supported by several studies) that PA contributes to health, there is little known in medical education about the modalities of the physical loads necessary to improve children’s immune systems and to protect children and adolescents from infections. This topic is especially important in the post-COVID-19 era, when organized physical activities restart worldwide. The resumption of normal life and of sport activities must be addressed, keeping in mind the indications and risks of a sudden increase in PA and sports, to avoid injuries and maximize the benefits. The availability of a variety of methods can be misleading, and it is necessary to select the appropriate method in order to boost the immune response. The methods of restarting PA after a prolonged rest in children are not well-known in their connections with the immune response, and therefore it is important to provide information about the different protocols of PA and their effects. There is currently a large body of knowledge on the topic even though existing studies on the efficacy of public health measures for the improvement of children’s health are sometimes limited in the studied cohorts [1] and in proposing applied solutions. This is especially true for children with special needs. It is also well-known that the secular trend toward a spread of obesity [2] in the developed countries, especially in the western world, has been further worsened by social isolation. Social factors pushing children to be sedentary [3] (e.g., mobile phone use, increased study pressure by the school system, and unavailability of cheap sports) have increased over the recent spread of COVID-19 and are linked to economic factors and limitations to social life. To provide effective physical activity (PA) programs, it is necessary to know the barriers to exercising from the social, psychological, and physiological perspectives and how they link to the cellular and systemic immunity. The aim of this integrative review is thus to analyze the available published knowledge concerning the connections between the immune system and the ways to recover physical functions from prolonged inactivity in children and adolescents.

## 2. Methods

Search strategy and selection criteria. This systematic review was performed according to the PRISMA guidelines [4,5]. Four databases were screened in December 2021/January 2022: PubMed, Cochrane Library, SPORTDiscus, and PsycINFO. Search terms were based on PICO standards [6] for children or adolescents of varying weight levels (normal weight, overweight, and obese) (subject population), different levels of spontaneous PA or PA/sport protocols (intervention), low levels of PA (comparator) and immune function (COVID-19) (outcome). A combination of MeSH terms, search terms in the title, abstract, relevant headings, and keywords, and synonyms for the search focus were employed. The search terms were: PA protocols and children, PA and children, children and exercise and immune system, children and exercise and leukocytes, children exercise and obesity and immune system, children, exercise and eosinophils, children and exercise and cytokines and inflammation, children and exercise and depression and immune system, children and exercise and TNF-α, sport and children and immune system, inflammation markers and exercise and children and sport, granulocytes, interferon y, monocytes, interleukins, NK and T-cells, helper T-cells, and different combinations of these terms. The inclusion criteria were peer-reviewed research papers or reviews, studies with more than 10 subjects, and studies published in English. The exclusion criteria were no controlled PA protocols (both spontaneous and organized), no peer-reviewed articles, no information on the content, and no mention of immunity. The protocol of this review was registered in the PROSPERO database for reviews (ID:313742).

Duplicates were manually removed.

## 3. Results

**Figure 1:**
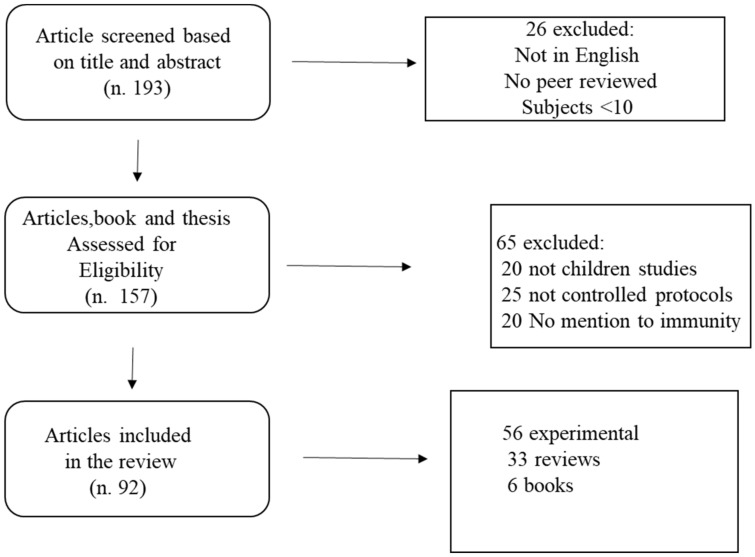
Flow chart of the search strategy.

A total of 193 papers met the search criteria. Of these, 157 were assessed for eligibility, and 92 met the inclusion criteria. The remaining studies were checked for eligibility. The search strategy flow chart is reported in Figure 1.

The results follow the main lines of research identified through the review. First, the evidence for the links between exercise, inflammatory processes, and the onset of fatigue is presented. Next, the immune response in children and its association with physical activity during the COVID-19 pandemic will be reviewed. Finally, a survey of proven methods of restoring basic motoric capacities will be presented.

### 3.1. Baseline Lactate, Blood Cells Counts, and Overweight

Lactate (La) is still the most-used metabolic marker of fatigue today. Knowing the La level in children is important to identify the dosage of the physical overload needed to elicit the appropriate training response. The maximal lactate steady state in prepubertal children (nine males and eight females, 9 to 13 years old) was measured on a cycloergometer as 4.1 ± 0.9 mmol L^−1^ [7], with no significant differences from adults: this allows the use of sustained aerobic efforts in children without the risk of excess lactate overload. It was found that the recovery time after the VO2 max testing was mostly dependent on individual characteristics (VO2 max) rather than age [8]. Therefore, the individual response is fundamental. Prepubertal children recover faster than untrained adults, are metabolically comparable to well-trained adult endurance athletes, and are less fatigued during high-intensity exercise than untrained adults [9]. Lactate was also shown to be dependent on body composition. The baseline lactate level in children 8–12 years old (50.7% females) was measured at 1.68 ± 0.07 mmol/L in children of normal weight, while in overweight and obese children, the baseline lactate level was measured at 2.11 ± 0.08 mmol/L [10] due to underlying systemic inflammation.

In the same study, the neutrophil and lymphocyte count changed by an estimate of 1300 cells/uL to 1600 cells/uL in post-pubertal boys (13–18 years old) after ten 2 min bouts of cycling at constant work rate at 65–75% of the halfway point between the anaerobic (lactate) threshold and the peak oxygen uptake. The observed changes were greater for males than they were for females, and they were greater for older children and adolescents than they were for younger [10]. When observing lactate levels, overweight and obese children showed greater levels of neutrophils and leukocytes both at baseline and after exercise. Maximal lactate levels were also measured in 15 male, 12-year-old prepubertal children (8.6 ± 1.6 mmol/L) and in 12 post-pubertal males (12.9 ± 0.9 mmol/L) with a Wingate test [11], and similar values were found in another study in a similar sample (10.7 ± 1.9 mmol/L). The same study confirmed that lactate recovery was not different from that of men (20 min La half-life) [12]. Overweight and obese children (defined as >85% weight percentiles for their age groups) also showed higher lymphocyte, leukocyte, neutrophile, and monocyte increases after exercise, in comparison to children of normal weight [13]. Generally, neutrophils increase acutely after various types of exercise [13]. Monocytes are of special interest since it has been observed that their presence, which is associated with inflammatory cytokines, results in tissue injury after exercise. One factor responsible for the activation of monocytes is leptin [14], a multifunctional hormone secreted by fat tissues, which is ubiquitous in the human body. Many studies exist on the association between leptin and monocytes in children, whereas the association between leptin and monocytes was observed in adult COVID-19 patients [14]. Leptin influences the bone marrow, instructing the hematopoietic niche cells and reducing the supply of inflammatory leukocytes [15]. Leptin is mobilized by exercise [15].

### 3.2. Immune System Response to Exercise

In comparison to normal weight children, overweight children (male, 10 years old) showed higher lymphocytes and a decreased T-cell percent [16] at rest after an exercise training. This result was obtained by providing a PA with a high motivational content (circus activities) that was physically challenging and, at the same time, enjoyable. The children practiced twice a week for 4.3 ± 0.5. The activities consisted of a 10 min warm-up with recreational and folklore dance followed by 40 min of solo acrobatics, with a combination of jumps, spins, and twists on an acrobatic trampoline; balancing on a wooden leg; and simple air acrobatics on a trapeze, rope, and platform. The training ended with 10 min of stretching exercises, and the total duration was 60 min at a mean heart rate of 185 bpm [16].

Even if the exercise is limited to a single bout, an acute effect on killer cells and T-cells can be observed [17,18] as was shown to increase after 260 stairs ascending at intensities > 75% of maximal oxygen uptake [18]. In adolescents (15.4 years old, 12 male and 23 females, all swimmers), the T regulatory cell count increased from 133 ± 11.2 to 196 ± 17.6 after a heavy training session consisting of swimming 440 yards at maximal intensity [17]. All the other markers of inflammation increased (leukocytes, lymphocytes, NK, B, T, T helper and suppressor, and FOXP3 (forkhead box Protein 3, a transcription factor that governs the maturation of Treg cells) [17].

Exhausting exercise and nutritional changes that occur in short term have been shown to be potentially damaging, likely because of the accumulation of too many reactive oxygen species during heavy efforts [19,20].

With respect to inflammation, some nutritional interventions have been recently discussed, even if clear conclusions have not been drawn about the effect of supplements in reducing inflammation and protection against COVID-19. One of the factors that was studied is vitamin D (contained in milk and derivates). It was shown that Vitamin D shut down the T cell-mediated inflammation [21].

Being overweight can also impair the cognitive development. Experimental results on mice showed that even the slow systemic inflammation caused by obesity was linked to the impairment of the hippocampal structures of the brain during the development [22]. Aerobic exercise ameliorates the brain structures and improves basic cognitive functions [23]. This effect on brain structures further reinforces the well-established association between obesity and anxiety/depression in children [24]. The ameliorating effects of exercise on depression seem to be at least partially mediated by immunomodulation. An association between a depression inventory test with IL-6 levels was found in depressed adolescents (18 males and 46 females, 15.6 years old) [25]. The same study observed a decline in IL-6 and TNF-α (tumor necrosis factor, responsible for cellular apoptosis) after 1 and 2 weeks of aerobic training, but no association of TNF-α was found to the depression scale.

A special population who needs care in the resumption of their normal life are children with cancer. Light to moderate exercise was observed to have an effect on cytokines’ expression and a positive impact in ameliorating the immune response, contrasted with the immunosuppression induced by chemotherapy. This effect was mainly observed as a resistance to upper respiratory tract infections [26]. 

An increase in neutrophil count only (not in eosinophil count) has been observed after an acute bout of exercise in children with acute lymphoblastoma [27]. 

Very few studies on young children exist. In a group of 5-year-old children who were very active (>9 h of activity declared by parents, reported by questionnaires), there was observed a lower secretion of IL-10, Il-13, insulin, GAD_65_, GAD_65_ -induced IL-5,CCL2, IL-6, Il-13, IFN-y, and TNF-α in comparison with low-activity children (<2 h/day in motion) [28]. The studies considering the acute effect of exercise on markers of immunity in young and prepubertal children are few and are reported in Table 1.

### 3.3. PA in COVID-19 Pandemic

COVID-19 itself, associated with forced inactivity, caused several important consequences in addition to the obvious decrease in lung function (a common feature of all respiratory illnesses). The most evident functional consequence of COVID-19 is probably the decrease in heart efficiency [30]. For athletes who had a COVID-19 infection, the European Association of Preventive Cardiology [30] recommends the gradual resumption of training after at least 7 days without any symptoms [31]. Further problems may occur in adolescents who train every day since the deprivation of exercise could lead to mental problems such as anxiety and depression [32]. 

An increase in spontaneous PA was observed when organized sport activities were not accessible. Children and adolescents increased their daily average spontaneous PA by 20 min per day in the 14–17-year-old age range, by 22 min in the 11–13-year-old age range, and by 47 min in 7–9-year-old age range. The highest increase in spontaneous PA was observed in 4–5-year-old children (57 min). These data show that children spontaneously increase their PA in an inversely proportional way to their age [33]. Spontaneous PA has been proposed to be centrally planned in the brain by unknown mechanisms that regulate the body’s thermogenesis [34]. These mechanisms likely reside in the crosstalk between the thermoregulation area and the motor area in the brain and are mediated by the pyrogenic activities of the cytokines produced during exercise, through the supraoptic nuclei and the hypothalamus [35].

## 4. Methods of Resuming Physical Activity

The general principles of training are well-known, and the methods used in sports can be adapted to exercise for health. Children have their own specificity. After menarche, girls start to significantly differ from boys in some physical performances. A paradigm of this differentiation is the standing long jump, which continues to increase after puberty in boys, while for the girls, it reaches a plateau [36]. This different progression in the development must be considered when planning the programs for PA resumption.

One way to restart exercising after periods of rest or reduced activity is to employ resistance training instead of aerobic conditioning. This type of exercise is better tolerated by obese children who feel uncomfortable with prolonged exertion [37]. A review of the available methods for PA resumption is presented in the following paragraphs.

### 4.1. Resistance Training

The variables in the design of a resistance training schedule include warm-up and cool-down, selection and order of exercise, intensity and volume, rest intervals between sets and exercises, and the repetition pace [37,38,39]. The warm-up is better tolerated if it is performed in a playful way, such as small-sided games with a ball [40,41]. Treadmill runs present some intrinsic risk for injury and are not recommended for children [42]. The scope of the warm-up is to increase the cardiorespiratory activity, improve the muscle capillarization, and activate the nervous system. Normally, the warm-up should not exceed 10 min. The order of the exercises follows the mass principle: first the bigger muscle masses, and then the smaller. Since the order of the exercises influences the perception of fatigue, it is better to perform the more fatiguing exercises first [43,44]. Therefore, legs are normally the first to be trained, followed by the upper limbs, and then the abdominal and trunk muscles. The exercises that respect the biomechanical principles should be preferred (e.g., proper limb alignment and physiological angles) over more complicated and potentially risky exercise [45]. Intensity is relatively easy to control when using resistance training machines. The initial load can be set at 10% of body weight, followed by a progression up to 70% bw, depending on age, sex, and physical fitness level [46]. Prolonged submaximal and maximal efforts are not recommended for a developing child. The simplest way to monitor the physical effort intensity is the heart rate, which should not be higher than 70% of the maximal heart rate, even if short bursts above this threshold are recommended to improve the heart’s contractile strength [47]. The load during the resistance training is organized in sets of repetitions. Five to ten repetitions of each set are recommended, with a rest interval of at least 2 min, allowing the refueling of the ATP–CP stores [48]. The total duration should not exceed 45 min/1 h of training, but it largely depends on individual physical capacity which, in turn, is affected by age, sex, and the previous level of training [46]. The pacing of the repetitions can be free-choice or imposed, using music or a metronome. The hypnagogic capacity of rhythm helps tolerate fatigue [49,50]. Every training session should end with a cool-down period, a series of low intensity activities able to deactivate the CNS (central nervous system). Stretching exercises are the most used cool-down activity. Two to four training units per week are recommended, and the literature suggests at least 2 months [46] of training to obtain an improvement. The effects of 8 weeks of training have been proven to last for an additional four weeks [51], following a 2/1 rules.

### 4.2. Aerobic Training

A light endurance training improves the CD4+/CD8+ ratio and the NK cell activity in obese children after 10 weeks [29]. In well-trained gymnasts and in untrained young females (aged 12 years), 20 min of intensive running (170/180 bpm) results in an acute significant elevation of T cell lymphocytes, T helpers, T suppressors, and natural killer cells that last 24 h. The increase in the leukocytes was caused by an increase in granulocytes, without any influence of the training status on these results [52].

Moderate aerobic activity can also prevent the detrimental effects of stress and depression due to the interactions between the neuroendocrine system and the immune system in the brain. Recent studies indicate the effect of the aerobic exercise on the brain is probably mediated by mTor (a protein kinase), as shown in rats’ studies [53]. Interestingly, regardless of whether spontaneous or controlled, the exercise increased the numbers of mTOR-positive glia in the brain structures of the striatum, hippocampus, and amygdala. However, a difference was observed in the effect of spontaneous versus controlled PA: the spontaneous exercise seemed to affect the astrocytes cells more, while the controlled exercise mostly affected the microglia cells in the inferior dentate gyrus [53].

It can be concluded that the exercise protocol and type are not important in determining the beneficial effects on mood, memory, and cognition in general, although there are differences in the brain structures affected by spontaneous or imposed PA.

The immune response benefits elicited by an intensive aerobic training (30 min of cycling per week at >70 <85% max heart rate) is highly individual-dependent. The difference in the individual response to aerobic loads is even higher in children who are immune-depressed [54]. A methodological consequence of this finding is that exercise intensity should be more carefully monitored in children who have undergone immunosuppressive therapies in comparison to healthy children [54]. However, the benefits of exercise on the mood and on the general health on this category of children are unquestionable [55].

Methodologically, aerobic exercises that have cognitive content are preferable over repetitive aerobic training due to their capacity to engage [56]. The use of motivating activities, for example, circus activities, offer a superior effect on mood and health [56]. Games have been shown to be superior to simple aerobic exercises in terms of cognitive and health outcomes and adherence [56]. However, a simple activity like stationary cycling is a choice when there are environmental constraints and stringent requirements for health and safety.

### 4.3. Flexibility Exercise and Mental Health

Yoga exercises have shown to be an effective way to manage anxiety in children [57,58]. Yoga exercises have thus been incorporated successfully in the physical education curricula of many schools and were welcomed with great enthusiasm by teachers [59]. Yoga exercises also have strong cultural and traditional content that reflects local cultural habits, and they are largely incorporated in the normal curricula in the schools of India [60]. Significant effects of yoga were observed in ameliorating the health status of children with special needs, such as the visually impaired [61], autistic [62], and those with Down syndrome [63]. In the 1970s following the wave of the Orient by the “hippies’ generation”, yoga became very popular in the Occident; and a method derived from yoga, the “stretching”, was developed, simplifying yoga postures and popularizing its use [64]. Bob Anderson’s famous book on stretching has sold nearly 3,5 million copies in 40 years [64].

A similar effect has been observed for tai chi, a traditional Chinese exercise method. Tai-chi has also been shown to reduce anxiety and improve the mood, both in children with special needs [65] and in regular school classes [66], and it is embedded in the physical education curricula in China. Recent studies on the effect of yoga during the COVID-19 pandemic show the efficacy of yoga and meditation in reducing anxiety and psychological distress in children [67]. The physiological underlying mechanisms of these slow-motion exercises seem to reside in the increased secretion of GABA (γ-aminobutyric acid) in the thalamic cells of the brain. After a 12-week, 60 min/week yoga training, the levels of GABA in children significantly increased in comparison to other subjects performing low intensity aerobic exercises (60 min walking on the treadmill at 4 km/h), with both of the activities performed at a metabolic equivalent of 3 METS [68]. The effect on the CPR (C-reactive protein) of those activities (enclosed qigong and meditation) has not been proven, and the literature reports mixed results [69]. An effect of these methods on IL-1, IL-6, and TNF-α was found in patients having previous high levels of these markers of inflammation [69]. A 16-week program of tai-chi was shown to reduce the Toll-like receptor (TLR)-4 stimulated production of IL-6 and TNF-α in monocytic populations, as compared to prior studies that used mixed mononuclear cell cultures or whole blood analysis [70]. Some more consistent effects of yoga, meditation, and tai-chi have been found in the expression of gene transcriptome pathways. It was observed that yoga reversed the pattern of leukocyte transcriptional alterations, including the activation of the genes regulated by the proinflammatory NF-κB/Rel family, a proinflammatory transcription factor [71].

Balance and coordinative exercises, which require a cognitive effort, must also be implemented gradually to avoid exhaustion and not be stressful. In fact, children who perceive themselves as less performant than their peers during physical education class or sport groups can manifest increased levels of anxiety and self-inadequacy and can encounter a burnout [72]. Thus, when working in groups, an approachable level of exercise difficulty is recommended.

Several strategies have been suggested to avoid the burnout caused by self-perceived motor incompetence. Cues for learning, goal setting, and exercise routines have been shown to be effective in preventing exhaustion [73]. From a social perspective, the main “actors” in preventing burnout in children are the parents: their pressure/anxiety around the child’s sport participation can be measured with appropriate scales [74].

## 5. Play and Games as Tools to Increase Physical Activity in Children

Social isolation is a limiting factor in the growth of children [75]. It can be countered by means of play. It is well-known that children’s involvement in physical activity is better obtained by means of playful activities. The literature on children playing is abundant [76], and play studies are a classic in the pedagogy studies [77]. Piaget [77], who is the scientist who likely performed the most extensive experimental studies on children’s games, observed that play provides an ideal solution for their own pleasure, helping them to reproduce and solve real-life conflicts and to ameliorate negative feelings. It also helps growth by structuring both the mental and physical boundaries of the human body, e.g., the notions of space and speed.

There are fewer studies about the efficacy of different playing conditions and/or the arrangements to increase children’s participation in PA [78]. However, some features of play seem determinant to involve children: be rewarding, interesting, and socially engaging. Most of the studies that tried to objectively measure play are based on questionnaires [79] or accelerometers [80]. All these studies agreed on the fact that most of the PA performed by children consists of free moderate to intense outdoors activities with a median amount of 40 min per day, at least in the developed countries, and in mild to cold environmental conditions [81]. Outdoor activities are beneficial to the immune system. The prolonged and gradual exposures to environmental factors provide a means to elevate resistance to allergies and infections [82]. However, even in more temperate climates such as south Italy, there is a lack of proper structures for children’s plays [83], not to mention the problems encountered by children with special needs, including the lack of affordability of the play structures [84,85] and the need for inclusive play settings [86], along with gender discrimination in certain cultures [87]. While spontaneous physical activity outside of school and sports clubs is prevalent, the benefits of stimulating less active children using guided play has been demonstrated to be useful [88]. Thus, play design is a crucial issue [89], as well the play’s affordance [90]. Play affordance has been defined as the possibility by less active children to participate in play, and it essentially depends on economic factors [90]. The theory of affordance [91] has identified eight characteristics of outdoor spontaneous playing, including safety, lighting, natural environment, affordability, challenges, connectivity, “character” or the overall perception of an outdoor place by children [90], and its usability [91].

A wise use of video games as motivation tools can improve the adherence, stimulate PA, and increase socialization [92].

A recent metanalytic study in children and adolescents (15,984 children 5 to 18 years old) showed that autonomous forms of motivation to exercise (i.e., intrinsic motivation) had a moderate, positive association with physical activity (*p* = 0.27 to 0.38). The study also found that controlled forms of motivation (i.e., introjection and external regulation) had weak, negative associations with physical activity (*p* = −0.03 to −0.17) as well as amotivation (*ρ* = −0.11 to −0.21). These results suggest that exercise is in some ways weakly influenced by motivation [93] but mostly emerges from spontaneous mechanisms. However, the authors observed that mostly of the surveyed studies presented some methodological problems, an issue previously known in the studies of motivation for PA [94]. The main problem seems to be the theoretical background that makes it difficult to define the construct of exercise adherence. In fact, intention can be transient and influenced by personal traits [94].

In summary, some general principles emerge from the literature that can be adopted in designing play programs to resume PA in children: first, the progressivity of physical loads [95]. Setting progressive dimensions of the playing field and giving time constrains are the simplest ways to determine the physical load of playing by using interesting activities [96]. The second principle is the variety of the movements allowed, which would exploit the physical potential of the participants. Proper recovery time is the third requisite for an effective activity reported in the literature [97]. All of these variables must be sized according to the target population.

## 6. Discussion

The literature on PA resumption after rest or illness is relatively abundant (e.g., bed rest studies [98]); however, there is little information about the underlying immune mechanisms that should guide the process of resuming PA in children. The literature indicates that the resumption of PA after periods of rest must follow a gradual pattern to avoid further problems. It also indicates that resistance, endurance, flexibility, and coordinative exercise are all suitable for boosting the immunity in children. Strength and endurance trainings provoke a direct effect on the increase in the immune defense of cells, while flexibility and relaxation exercises act to reduce stress and increase the internal secretion of relaxing hormones. Several positive effects of PA in stimulating immunity are well-established and are consistent in both healthy and ill children.

Overall, the stimulation of immune responses must follow a gradual increase in loads, an interesting approach, and an appropriate stimulation. The emergence of the pandemic has shown the importance of PA in increasing the immune defenses and the necessity to implement large-scale plans for the resumption and the diffusion of PA in children, guided by scientific principles. These actions can be only realized with a public intervention aiming to restore the sport clubs damaged by the lockdowns and also to provide through educational plans for professional involvement. As Sigmund Freud [98] stated, the man tends to the inorganic, meaning that sedentarism fosters other sedentarism. Measures to counteract the inactivity by stimulation are urgent, especially after times of prolonged rest associated with sedentarism.

## 7. Conclusions

Some directions on children’s exercises to resume PA and to boost the immune system can be drawn from the literature. PA must be gradual and progressive in load and intensity and must be adapted to the child’s health status and body size, with a preference of non-loading activities for overweight children. Mid-intensity effort should be preferred, with some peak intensity, and limited by time. Exercise must be motivating and mentally stimulating. When possible, games should be used, but more controlled exercise settings are necessary for children with special needs. It has been shown that when structured exercise (e.g., sport participation) is impossible, natural PA emerges spontaneously and is of benefit as well. Individualized schedules are necessary since the responses are highly individual and do not always depend on controllable internal and environmental factors, especially in children with special needs. Regular participation in exercise class and sports is an indispensable tool to maintain health, to improve the mood, to reduce inflammation, and to boost the immune system in children and adolescents. Additionally, this review evidenced that when there is a lack of organized/sport activities, PA emerges spontaneously. This fact opens the door for new research questions: if healthy children must be left free to move at their choice, what amount must be geared toward organized physical activity, and what amount is the optimal balance.

An understanding of the methodological and environmental tools that can be employed to increase PA in children is essential for facing the resumption of PA after periods of problematic social changes. A limitation of this review is the inclusion of a relatively low number of studies considering the basic molecular mechanism of the immune response in children. However, the relationships between the social factors and the immune responses are still being investigated. A hypothesis for further studies can be directed toward exploring the interaction between the social constrains of exercising, the physiological effects of exercising, and the immune response to exercise.

## Figures and Tables

**Table 1:** Summary of the studies reporting acute effect of exercise on markers of immunity in children.

Author	Year	Subject Details	Intervention	Markers	Outcomes
Chen et al. [10]	2018	15 total, male, 12 years old	cycloergometer 75%	leukocytes	increased
		12 total, male, 16 years old	20 min interval training		
Wilson et al. [17]	2009	35 total swimmers, 12 males, 15 years old	7 min max swimming	leukocytes	increased
				lymphocytes	increased
				N, T, K cells	increased
				Treg	increased
				FOXP3	increased
Wunram et al. [25]	2021	46 females and 18 males, 15 years old	vibration training and	Tnfα	decreased
		depressed	aerobic training	IL-6	decreased
Carlsson et al. [28].	2016	5 years old, high level of PA	spontaneous PA	IL-6	decreased
		high level of PA		IL-10	decreased
				IL-13	decreased
				Tnfα	decreased
				IFN-γ	decreased
				insulin	decreased
Eliakim et al. [29]	1997	female, 10–12 years old			
		7 gymnasts, 6 untrained	20 min running at 170/180 bpm	T, T h, T s	increased
				B	no effect
				Ig a	no effect
				Ig m	no effect
				Ig e	no effect
				Ig g	no effect

## Data Availability

Not applicable.
